# INSIDE THE BLACK BOX OF MANAGED CARE: SERVING AT-RISK OLDER ADULTS IN MASSACHUSETTS’ SENIOR CARE OPTIONS PROGRAM

**DOI:** 10.1093/geroni/igae098.3395

**Published:** 2024-12-31

**Authors:** Pamela Nadash, Marc Cohen, Edward Miller, Janelle Fassi, Maryssa Pallis, Megan Siebecker, Elizabeth Simpson

**Affiliations:** University of Massachusetts Boston, Boston, Massachusetts, United States; University of Massachusetts Boston, Boston, Massachusetts, United States; University of Massachusetts Boston, Boston, Massachusetts, United States; University of Massachusetts Boston, Goffstown, New Hampshire, United States; University of Massachusetts Boston, Boston, Massachusetts, United States; University of Massachusetts Boston, Boston, Massachusetts, United States; University of Massachusetts Boston, Boston, Massachusetts, United States

## Abstract

People dually eligible for Medicare and Medicaid are at risk for poorly coordinated care, high-cost health service utilization, and poor outcomes. Commonwealth Care Alliance (CCA), a not-for-profit managed care organization, serves approximately 35,000 people dually eligible for Medicare and Medicaid in the Commonwealth of Massachusetts. CCA has two primary products: Senior Care Options, which has served people 65 years or older since 2003, and One Care, which has served people 18-64 years of age since 2013. Drawing from key informant interviews and other data collection, this study sought to understand the organizational experience of the CCA model. It identifies the challenges CCA faces in ensuring that its vulnerable membership has access to both appropriate clinical care and the broad range of health-related services that are critical to the health of members. It also identifies the plan’s mechanisms for addressing these challenges. The evolution of CCA’s model of care and the various mechanisms it adopted over time to adapt to the needs of its membership and comply with state and federal requirements are discussed. These changes are then placed in the context of ongoing conversations about the most effective models of integrated care. Findings highlight CCA’s focus on health-related social needs; the impact of member advocacy on program design; and the implications of non-profit status for workplace culture and resources.

